# Synthesis of Highly Substituted Oxazoles through Iodine(III)-Mediated Reactions of Ketones with Nitriles

**DOI:** 10.3390/molecules170911046

**Published:** 2012-09-13

**Authors:** Akio Saito, Nao Hyodo, Yuji Hanzawa

**Affiliations:** Laboratory of Organic Reaction Chemistry, Showa Pharmaceutical University, 3-3165 Higashi-tamagawagakuen, Machida, Tokyo 194-8543, Japan; Email: a06097@ug.shoyaku.ac.jp

**Keywords:** single-step synthesis, iodine(III), oxazole, ketone, nitrile

## Abstract

In the presence of trifluoromethanesulfonic acid (TfOH) or bis(trifluoromethane-sulfonyl)imide (Tf_2_NH), iodosobenzene (PhI=O) efficiently promoted the reactions of dicarbonyl compounds as well as monocarbonyl compounds with nitriles to give 2,4-disubstituted and 2,4,5-trisubstituted oxazole in a single step under the mild conditions.

## 1. Introduction

The synthesis of oxazole compounds has attracted a great deal of attention due to the widespread application of oxazole derivatives in biologically active compounds as well as versatile building blocks in organic synthesis [1,2,3]. For the purpose of synthesizing highly substituted oxazole compounds, an intramolecular reaction, the so-called Robinson-Gabriel cyclocondensation of α-acylamino ketones in the presence of dehydrating reagents [H_2_SO_4_, POCl_3_, (CF_3_SO_2_)_2_O, and so on] has been commonly employed [4,5,6]. As for the intermolecular approaches to highly substituted oxazoles, α-diazo ketones [7,8,9], α-halo ketones [10,11], α-sulfonyloxy ketones [12], and iodonium ylides of ketones [13] have been used as a reactive synthetic intermediate. The preparation of α-acylamino ketones or these intermediates, however, requires a multi-step synthesis and/or the harsh reaction conditions. Although the direct synthesis of oxazoles from simple ketones and nitriles with oxidants based on Tl(III) [14], Hg(II) [15], Fe(III) [16], or Cu(II) [17] have been developed as a convenient procedure, these procedures have met with the drawbacks including the limitation of the substrates and/or the use of toxic oxidants.

Hypervalent iodine(III) reagents have gained increasing popularity in organic syntheses due to their low toxicity, mild reactivity, high stability, easy handling, and so on [18]. Among them, [hydroxyl-(tosyloxy)iodo]benzene (Koser’s reagent) and related reagents have been reported to work well for the α-sulfoxylations of ketones [19,20]. [Hydroxy(2,4-dinitrobenzenesulfonyloxy)iodo]benzene (HDNIB) has been used in the stepwise and one-pot synthesis of oxazoles via the formation of α-sulfonyloxy ketones as intermediate from simple ketones [21]. In addition, phenyliodine(III) diacetate (PIDA) with trifluoromethanesulfonic acid (TfOH) [22] or the iodoarene-oxone-TfOH system [23] efficiently promoted the direct synthesis of oxazoles from monocarbonyl compounds, such as alkyl aryl ketones with nitriles. To the best of our knowledge, however, there is no report about the direct synthesis of oxazoles from simple dicarbonyl compounds and nitriles [21,24,25]. As a part of our study on the iodine(III)-mediated synthesis of heterocycles [26,27], we carried out research on iodine(III) reagents for the direct synthesis of oxazoles from monocarbonyl or dicarbonyl compounds. In this article, we describe a single-step synthesis of highly substituted oxazoles by the reaction of ketones with nitriles in the presence of iodosobenzene (PhI=O) and a Brønsted acid.

## 2. Results and Discussion

### 2.1. Evaluation of Oxidants for the Direct Synthesis of Oxazole

At the outset, we focused on the investigation of reactive oxidants for the reaction of monocarbonyl compounds with nitriles as shown in Table 1. In the presence of PIDA (1.2 equiv.) with TfOH (4.5 equiv.), the reaction of acetophenone (**1a**) in acetonitrile (MeCN) afforded the oxazole **2a** in 94% yield at 80 °C for 2 h (entry 1) [23], albeit low yield (22%) at ambient temperature for 3 h (entry 2). Exploring milder conditions, we examined the oxidants which were prepared from miscellaneous Brønsted acids (1.5–3.0 equiv.) and PhI=O (1.5 equiv.) [28,29], in the reaction of **1a** in MeCN (entries 3–8). Although the use of *p*-toluenesulfonic aicd (TsOH) gave α-tosyloxy ketone **3** as a main product (entry 3), the other acids led to the desired formation of oxazole compound **2a** (entries 4–8). Thus, 3.0 equiv. TfOH showed the similar results to entry 1 (entry 7) and 3.0 equiv. bis(trifluoromethane-sulfonyl)imide (Tf_2_NH) produced a good yield of **2a** (86%) after 3 h at ambient temperature (entry 9). It should be mentioned that the use of 10 equiv. of MeCN in 1,2-dichloroethane instead of MeCN solvent decreased the yield of **2a**, even under the similar conditions mediated by PhI=O with TfOH or Tf_2_NH.

For the formation of oxazole **5a** from β-keto ester **4a** in MeCN, PhI=O/Tf_2_NH system turned out to display superior activity to iodine(III) reagents/TfOH (Table 2). Thus, PhI=O (1.5 equiv.) with Tf_2_NH (3.0 equiv.) improved the yield of **5a** to 51% at 80 °C for 16 h (entry 4), compared to the use of 1.5 equiv. PIDA or 1.5 equiv. PhI=O in the presence of TfOH (3.0–4.5 equiv.), in which **4a** were converted to **5a** in only 4–7% yields even at 80 °C for 24–25 h (entries 1 and 2). In the case of the extension of the reaction time (72 h) or the increase in the amount of Tf_2_NH (6.0 equiv.), **5a** was obtained in 79–81% yields (entries 5 and 6).

**Table 1:** Evaluation of oxidants for the reaction of acetophenone (**1a**) in MeCN.

Entry	Oxidant (equiv)	Acid (equiv.)	Temp. (°C)	Time (h)	2a (%) ^[a]^	
1 ^[b]^	PIDA (1.2)	TfOH (4.5)	80	2	94	
2	PIDA (1.2)	TfOH (4.5)	rt	3	22	
3	PhI=O (1.5)	TsOH (1.5)	80	18	-	( **3** 69)
4	PhI=O (1.5)	HBF_4_/Et_2_O (1.5)	80	18	54	
5	PhI=O (1.5)	TfOH (1.5)	80	18	69	
6	PhI=O (1.5)	Tf_2_O (1.5)	80	18	21 ^[c]^	
7	PhI=O (1.5)	TfOH (3.0)	80	3	94	
8	PhI=O (1.5)	Tf_2_NH (1.5)	80	3	40	( **1a** 16)
9	PhI=O (1.5)	Tf_2_NH (3.0)	rt	3	86	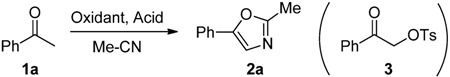

^[a]^ Yields were determined by ^1^H-NMR analysis; ^[b]^ Ref. [21]; ^[c]^ 2,4-Dimethyl-6-phenylpyrimidine was obtained in 38% yield.

**Table 2:** Evaluation of oxidants for the reaction of benzoylacetate **4a** in MeCN.

Entry	Oxidant	Acid		Time (h)	5a (%) ^[a]^
1	PIDA	TfOH ^[b]^		24	4
2	PhI=O	TfOH		25	7
3	PhI=O	TfOH		115	41
4	PhI=O	Tf_2_NH		16	51
5	PhI=O	Tf_2_NH		72	79
6	PhI=O	Tf_2_NH ^[c]^		3	81 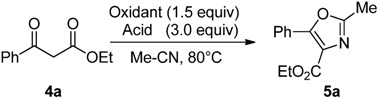

^[a]^ Yields were determined by ^1^H-NMR analysis; ^[b]^ TfOH: 4.5 equiv.; ^[c]^ TfOH: 6.0 equiv.

### 2.2. Scope of the Direct Synthesis of Oxazoles Using PhI=O with TfOH or Tf_2_NH

**Scheme 1:**
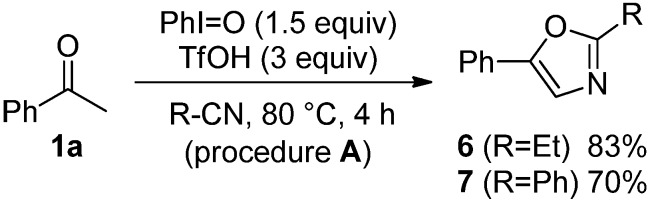
The reactions of **1a** in EtCN or PhCN by the means of procedure **A**.

The scope of monocarbonyl compounds **1** and dicarbonyl compounds **4** by means of the PhI=O (1.5 equiv.)-mediated procedure **A** (acid: 3.0 equiv. TfOH), **B** (acid: 3.0 equiv. Tf_2_NH), or **C** (acid: 6.0 equiv. Tf_2_NH) was shown in Table 3 and Table 4 and Scheme 1. Procedure **A** could be applied to the reactions of monocarbonyl compounds **1a**–**g** in MeCN, and the corresponding oxazoles **2a**–**g** were obtained in 53–94% yields at 80 °C (Table 3, entries 1, 3–6, 8, and 9). Furthermore, procedure **B** brought about the formation of **2a** or **2e** at ambient temperature (entries 2 and 7). In the case of benzoylacetonitrile (**1g**), an increase in the amount of Tf_2_NH (procedure **C**) improved the yield of **5f** up to 69% at ambient temperature for 24 h (entry 11). Although the dicarbonyl compounds **4a**,**b** required the long time (72–139 h) to give good yields of products through procedure **B** (entries 12 and 14), procedure **C** reduced the reaction times (3 h) giving rise to **5a**–**c** in 67–83% yields (entries 13, 15 and 17). Bicyclic oxazole **5d** could be formed by procedure **C**, albeit in only 40% yield after 167 h (entry 18). Unfortunately, *p*-methoxyacetophenone and 4-phenyl-2-butanone did not give the desired products with any of the procedures. By procedure **A**, the reaction of acetophenone (**1a**) in propionitrile (EtCN) or benzonitrile (PhCN) instead of MeCN smoothly proceeded at 80 °C for 4 h to yield the corresponding oxazole **6** or **7** in good yields (Scheme 1). The procedure **C** could be applied to the reaction of dicarbonyl compounds **4a**–**c** in EtCN or PhCN (Table 4).

**Table 3:** The reactions of **1** or **4** in MeCN by the means of procedures A, B, or C ^[a]^.

Entry	1 or 4	R^1^	R^2^	Procedure	(°C)	(h)	2 or 5	Yield (%) ^[b]^
1	**1a**	Ph	H	**A**	80	3	**2a**	88
2	**1a**			**B**	rt	3	**2a**	86
3	**1b**	*m*-Me-C_6_H_4_	H	**A**	80	3	**2b**	75
4	**1c**	*p*-Cl-C_6_H_4_	H	**A**	80	3	**2c**	86
5	**1d**	*p*-NO_2_-C_6_H_4_	H	**A**	80	3	**2d**	73
6	**1e**	Ph	Me	**A**	80	3	**2e**	94
7	**1e**			**B**	rt	2	**2e**	91
8	**1f**	Ph	Cl	**A**	80	49	**2f**	68
9	**1g**	Ph	CN	**A**	80	20	**2g**	53
10	**1g**			**B**	80	20	**2g**	38
11	**1g**			**C**	rt	24	**2g**	69
12	**4a**	Ph	OEt	**B**	80	72	**5a**	78
13	**4a**			**C**	80	3	**5a**	81
14	**4b**	Ph	Ph	**B**	80	139	**5b**	89
15	**4b**			**C**	80	3	**5b**	83
16	**4c**	Me	Me	**B**	80	120	**5c**	35
17	**4c**			**C**	80	3	**5c**	67
18	**4d**	-(CH_2_)_4_-		**C**	80	167	**5d**	40 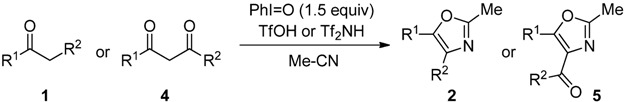

^[a]^ Procedure **A**: 3 equiv. TfOH was used as a Brønsted acid. Procedure **B**: 3 equiv. Tf_2_NH was used as a Brønsted acid. Procedure **C**: 6 equiv. Tf_2_NH was used as a Brønsted acid; ^[b]^ Isolated yields.

**Table 4:** The reactions of **4a**–**c** in EtCN or PhCN by the means of procedure **C**.

Entry	4	R^1^	R^2^	R^3^	(h)	2 or 5	Yield (%) ^[a]^
1	**4a**	Ph	OEt	Et	2	**8a**	83
2	**4a**			Ph	3	**9a**	72
3	**4b**	Ph	Ph	Et	3	**8b**	89
4	**4b**			Ph	3	**9b**	67
5	**4c**	Me	Me	Et	20	**8c**	56
6	**4c**			Ph	20	**9c**	60 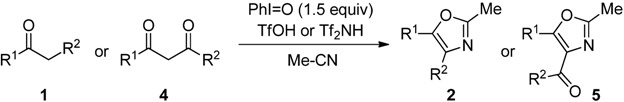

^[a]^ Isolated yields.

### 2.3. Mechanistic Considerations

**Scheme 2:**
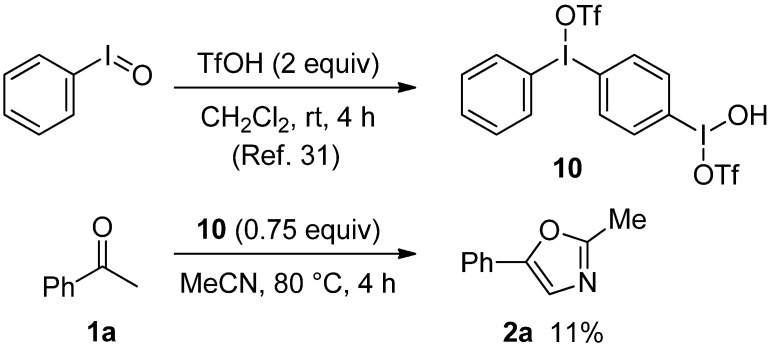
Preparation of **10** and the reaction of **1a** with **10**.

Since it has been known that PhI=O reacts with two equiv. of TfOH in CH_2_Cl_2_ to produce to the oxidant **10** [30], to better understand the present oxazole formation, we examined the reaction of acetophenone (**1a**) with **10** (Scheme 2). Thus, under conditions similar to those of entry 7 in Table 1, **1a** was treated with **10** (0.75 equiv.) instead of PhI=O (1.5 equiv.) and TfOH (3 equiv.) in MeCN to give **2a** in only 11% yield at 80 °C for 3 h. Therefore, **10** would not be considered to take part in the present oxazole formation. 

**Scheme 3:**
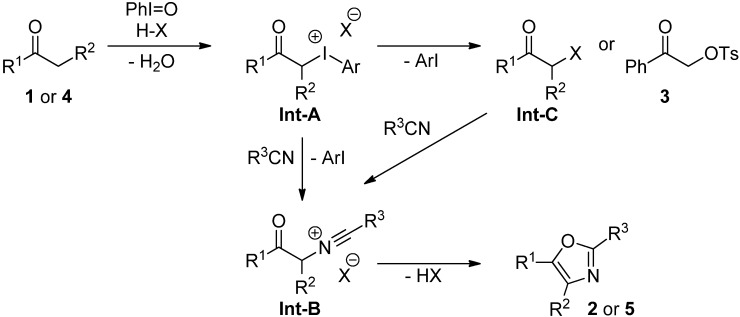
Proposed reaction mechanism of direct synthesis of oxazoles.

On the basis of abovementioned observations and the previous reports about the iodine(III)-mediated synthesis of oxazoles [21,22,23], the mechanism for the present oxazole formation from ketones **1** or **4** with nitriles as shown in Scheme 3 is proposed. That is, α-iodanyl ketone **Int-A**, which is generated from **1** or **4** with PhI=O and H-X (TfOH or Tf_2_NH), would be converted to **Int-B** by the Ritter-type reaction with R^3^CN. **Int-C** generated by the reductive elimination of ArI might also be a possible intermediate for the formation of **Int-B**, and the subsequent cyclization of **Int-B** gives oxazoles **2** or **5**. The formation of **Int-A** and/or **Int-C** is supported by the formation of α-tosyloxy ketone **3** (69%) in the case of TsOH as an acid (Table 1, entry 3) [19,23].

## 3. Experimental

### 3.1. General

All starting materials and reagents were commercially available. Dried organic solvents were purchased and used without further drying. Unless otherwise stated, all reactions were conducted under an argon atmosphere. Melting points were measured on a Yanaco SP-M1 melting point apparatus (Yanagimoto Co.) and were uncorrected. IR spectra were recorded on a HORIBA FT-710 FT-IR spectrometer. ^1^H and ^13^C-NMR spectra were measured in CDCl_3_ with a Bruker AV300M FT NMR spectrometer at 300 and 75 MHz, and the chemical shifts are given in ppm using CHCl_3_ (7.26 ppm) for ^1^H-NMR and CDCl_3_ (77.0 ppm) for ^13^C-NMR as an internal standard, respectively. Mass spectra and HRMS were recorded by FAB method on a JMS-HX110 Mass spectrometer. Elemental analysis was measured on a Perkin-Elmer 240B or Elemental Vavio EL. For the TLC analysis, Merck precoated TLC plates (silica gel 60 F_254_) were used. Column chromatography was performed on Silica gel 60N (63–200 μm, Kanto Kagaku Co., Ltd.).

### 3.2. General Procedure for the Iodine(III)-Mediated Synthesis of Oxazoles

Ketone **1** or **4** (0.4 mmol) was added to a solution of iodosobenzene (132 mg, 0.6 mmol) and trifluoromethanesulfonic acid (106 μL, 1.2 mmol) or bis(trifluoromethanesulfonyl)imide (337 or 675 mg, 1.2 or 2.4 mmol), which were premixed in acetonitrile, propionitrile, or benzonitrile (2 mL) at 0 °C for 5 min, and the reaction mixture was stirred at ambient temperature or 80 °C until the consumption of substrate by TLC analysis. The mixture was diluted with ether and filtered through a short alumina column. After concentration of the filtrate to dryness, the subsequent purification gave the corresponding oxazole **2** or **5**. **2a** [21], **2c** [21], **2d** [31], **2e** [21], **6** [21], **7** [32], and **9a**–**c** [33] were identified by the comparison with ^1^H-NMR spectra reported in the appropriate literature.

*2-Methyl-5-(3′-methylphenyl)oxazole* (**2b**). Colorless oil. IR (neat) ν cm^−1^; 3054, 2925, 2863, 1610, 1560, 1519, 784, 748. ^1^H-NMR (CDCl_3_) δ 2.38 (s, 3H), 2.51 (s, 3H), 7.02 (d, *J* = 7.6 Hz, 1H), 7.18 (s, 1H), 7.28 (t, *J* = 7.6 Hz, 1H), 7.40 (d, *J* = 9.2 Hz, 2H). ^13^C-NMR (CDCl_3_) δ 14.0, 21.4, 121.0, 121.6, 124.5, 128.0, 128.7, 128.9, 138.5, 151.2. FAB-LM *m/z*: 174.2 (M^+^+H). FAB-HM Calcd for C_11_H_12_NO: 174.0919, Found: 174.0906.

*4-Chloro-2-methyl-5-phenyloxazole* (**2e**). White solid. Mp 56 °C. IR (KBr) ν cm^−1^; 3050, 3002, 2923, 2856, 1438, 1378. ^1^H-NMR (CDCl_3_) δ 2.50 (s, 3H), 7.30–7.36 (m, 1H), 7.41–7.46 (m, 2H), 7.83–7.84 (m, 2H). ^13^C-NMR (CDCl_3_) δ 14.2, 124.8, 127.0, 128.7, 143.8, 159.4. FAB-LM *m/z*: 194.1 (M^+^+H). FAB-HM Calcd for C_10_H_9_ClNO: 194.0373, Found: 194.0383. Anal. Calcd for C_10_H_8_ClNO: C, 62.03; H, 4.06; N, 7.23. Found: C, 62.21; H, 4.47; N, 7.23.

*4-Cyano-2-methyl-5-phenyloxazole* (**2f**). White solid. Mp 49 °C. IR (KBr) ν cm^−1^; 3062, 2927, 2854, 2225. ^1^H-NMR (CDCl_3_) δ 2.56 (s, 3H), 7.48–7.50 (m, 3H), 7.89–7.92 (m, 2H). ^13^C-NMR (CDCl_3_) δ 13.9, 108.0, 113.8, 125.2, 125.5, 129.3, 130.9, 157.8, 161.1. FAB-LM *m/z*: 185.2 (M^+^+H). Anal. Calcd for C_11_H_8_N_2_O: C, 71.73; H, 4.38; N, 15.21. Found: C, 71.91; H, 4.39; N, 15.02.

*4-Ethoxycarbonyl-2-methyl-5-phenyloxazole* (**5a**). White solid. Mp 64 °C. IR (KBr) ν cm^−1^; 3054, 3006, 2977, 2927, 2857, 1598, 1560, 1488. ^1^H-NMR (CDCl_3_) δ 1.38 (t, *J* = 7.1 Hz, 3H), 2.54 (s, 3H), 4.40 (q, *J* = 7.1 Hz, 2H), 7.43–7.45 (m, 3H), 8.02–8.05 (m, 2H). ^13^C-NMR (CDCl_3_) δ; 13.9, 14.3, 61.3, 106.9, 127.1, 128.3, 128.3, 130.1, 155.3, 159.9, 162.2. FAB-LM *m/z*: 232.2 (M^+^+H). FAB-HM Calcd for C_13_H_14_NO_3_: 232.0974, Found: 232.0977. Anal. Calcd for C_1__3_H_13_NO_3_: C, 67.52; H, 5.67; N, 6.06. Found: C, 67.80; H, 5.81; N, 5.89.

*4-Benzoyl-2-methyl-5-phenyloxazole* (**5b**). Colorless oil. IR (neat) ν cm^−1^; 3031, 2965, 2927, 2927, 2856, 1710. ^1^H-NMR (CDCl_3_) δ 2.58 (s, 3H), 7.40–7.47 (m, 5H), 7.47–7.55 (m, 1H), 7.95–7.98 (m, 2H), 8.05–8.08 (m, 2H). ^13^C-NMR (CDCl_3_) δ; 13.8, 127.3, 127.6, 128.1, 128.4, 130.0, 130.2, 132.9, 133.7, 137.5, 154.7, 159.0. FAB-LM *m/z*: 264.2 (M^+^+H). FAB-HM Calcd for C_1__8_H_14_NO_2_: 264.1025, Found: 264.1014. Anal. Calcd for C_17_H_13_NO_2_: C, 77.55; H, 4.98; N, 5.32. Found: C, 77.38; H, 5.16; N, 5.40.

*4-Acetyl-2,5-dimethyloxazole* (**5c**). White solid. Mp 39 °C. IR (KBr) ν cm^−1^; 1681. ^1^H-NMR (CDCl_3_) δ 2.73 (s, 3H), 2.43 (s, 3H), 2.50 (s, 3H). ^13^C-NMR (CDCl_3_) δ 12.0, 13.6, 27.8, 134.5, 154.2, 158.4, 194.7. EI-LM *m/z*: 139.1 (M^+^). Anal. Calcd for C_7_H_9_NO_2_: C, 60.42; H, 6.52; N, 10.07. Found: C, 60.02; H, 6.16; N, 9.82.

*6,7-Dihydro-2-methylbenzo[d]oxazole-4(5H)-one* (**5d**). Colorless oil. IR (neat) ν cm^−1^; 2952, 2927, 2854, 1682. ^1^H-NMR (CDCl_3_) δ 2.12–2.20 (m, 2H), 2.52 (s, 3H), 2.57 (t, *J* = 5.9 Hz, 2H), 2.81 (t, *J* = 6.1 Hz, 2H). ^13^C-NMR (CDCl_3_) δ 14.4, 23.2, 23.8, 38.0, 144.2, 156.1, 165.9, 185.8. FAB-HM Calcd for C_8_H_10_NO_2_: 152.0712, Found: 152.0697.

*4-Ethoxycarbonyl-2-ethyl-5-phenyloxazole* (**8a**). White solid. Mp 37 °C. IR (KBr) ν cm^−1^; 3066, 2983, 2933, 2875, 1714, 1240, 1189. ^1^H-NMR (CDCl_3_) δ 1.38 (t, *J* = 7.1 Hz, 3H), 1.39 (t, *J* = 7.6 Hz, 3H), 2.88 (q, *J* = 7.6 Hz, 2H), 4.40 (q, *J* = 7.1 Hz, 2H), 7.42–7.47 (m, 3H), 8.01–8.04 (m, 2H). ^13^C-NMR (CDCl_3_) δ 11.2, 14.3, 21.6, 61.3, 126.7, 127.1, 128.3, 130.0, 155.0, 162.2, 164.1. FAB-LM *m/z*: 264.2 (M^+^+H). FAB-HM Calcd for C_14_H_16_NO_3_: 246.1130, Found: 246.1126. Anal. Calcd for C_14_H_15_NO_3_: C, 68.56; H, 6.16; N, 5.71. Found: C, 68.79; H, 6.06; N, 5.61.

*4-Benzoyl-2-ethyl-5-phenyloxazole* (**8b**). Colorless oil. IR (neat) ν cm^−1^; 3064, 2981, 2937, 1656. ^1^H-NMR (CDCl_3_) δ 1.43 (t, *J* = 7.6 Hz, 3H), 2.92 (q, *J* = 7.6 Hz, 2H), 7.40–7.45 (m, 5H), 7.53–7.58 (m, 1H), 7.94–7.97 (m, 2H), 8.06–8.10 (m, 2H). ^13^C-NMR (CDCl_3_) δ 11.3, 21.7, 127.5, 127.7, 128.2, 128.5, 130.0, 130.4, 133.0, 133.7, 137.5, 154.5, 163.4. FAB-LM *m/z*: 278.2 (M^+^+H). FAB-HM Calcd for C_18_H_16_NO_2_: 278.1181, Found: 278.1177. Anal. Calcd for C_18_H_15_NO_2_: C, 77.96; H, 5.45; N, 5.05. Found: C, 77.95; H,5.42; N,5.10.

*4-Acetyl-2-ethyl-5-menyloxazole* (**8c**). Colorless oil. IR (neat) ν cm^−1^; 1685. ^1^H-NMR (CDCl_3_) δ 1.25 (t, *J* = 7.6 Hz, 3H), 2.42 (s, 3H), 2.50 (s, 3H), 2.68 (q, *J* = 7.6 Hz, 2H). ^13^C-NMR (CDCl_3_) δ; 11.1, 12.1, 21.4, 27.8, 134.4, 154.0, 162.7, 194.9. FAB-LM *m/z*: 154.1 (M^+^+H). FAB-HM Calcd for C_8_H_12_NO_2_: 154.0868, Found: 154.0873. 

### 3.3. Formation of α-Tosyloxy Ketone ***3*** under the Iodine(III)-Mediated Conditions

Ketone **1a** (47 μL, 0.4 mmol) was added to a solution of iodosobenzene (132 mg, 0.6 mmol) and *p*-toluenesulfonic acid monohydrate (114 mg, 0.6 mmol), which were premixed in MeCN (2 mL) at 0 °C for 5min, and the reaction mixture was stirred at 80 °C for 18 h. The mixture was diluted with ether and filtered through a short alumina column. After concentration of the filtrate to dryness, the subsequent purification gave **3** (73.2 mg, 0.25 mmol, 63%). Compound **3** was identified by the comparison with the ^1^H-NMR spectrum reported in the literature [20].

## 4. Conclusions

We have demonstrated the single-step synthesis of highly substituted oxazoles from ketones and nitriles by the use of iodosobenzene with trifluoromethanesulfonic acid or bis(trifluoromethane-sulfonyl)imide. The present procedure could be applied not only to monocarbonyl compounds, but also to dicarbonyl ones. In particular, we believe that the reactivity of iodosobenzene with bis(trifluoro-methanesulfonyl)imide sheds light on a new possibility for the use of hypervalent iodine compounds in organic synthesis.
